# DNA Polymerase I Large Fragment from *Deinococcus radiodurans*, a Candidate for a Cutting-Edge Room-Temperature LAMP

**DOI:** 10.3390/ijms25031392

**Published:** 2024-01-23

**Authors:** Marilena Manzo, Assunta Serra, Emilia Pedone, Luciano Pirone, Viviana Scognamiglio, Mariarita De Felice, Mariarosaria De Falco

**Affiliations:** 1 Institute of Bioscience and BioResources, National Research Council, Via Pietro Castellino 111, 80131 Naples, Italy; 2 Institute of Biostructures and Bioimaging, National Research Council, Via Pietro Castellino 111, 80131 Naples, Italy; 3 Institute of Crystallography, National Research Council, Department of Chemical Sciences and Materials Technologies, Via Salaria km 29.300, Monterotondo, 00015 Rome, Italy

**Keywords:** DNA replication, DNA polymerase, LAMP, extremophiles, *Deinococcus radiodurans*

## Abstract

In recent years, the loop-mediated isothermal amplification (LAMP) technique, designed for microbial pathogen detection, has acquired fundamental importance in the biomedical field, providing rapid and precise responses. However, it still has some drawbacks, mainly due to the need for a thermostatic block, necessary to reach 63 °C, which is the BstI DNA polymerase working temperature. Here, we report the identification and characterization of the DNA polymerase I Large Fragment from *Deinococcus radiodurans* (DraLF-PolI) that functions at room temperature and is resistant to various environmental stress conditions. We demonstrated that DraLF-PolI displays efficient catalytic activity over a wide range of temperatures and pH, maintains its activity even after storage under various stress conditions, including desiccation, and retains its strand-displacement activity required for isothermal amplification technology. All of these characteristics make DraLF-PolI an excellent candidate for a cutting-edge room-temperature LAMP that promises to be very useful for the rapid and simple detection of pathogens at the point of care.

## 1. Introduction

DNA polymerases are ubiquitous enzymes that replicate DNA in all living cells. A large number of DNA polymerases from various organisms have been identified and characterized since their first discovery from *Escherichia coli* in the 1950s by Arthur Kornberg [1]. This DNA polymerase was later classified as DNA polymerase I, which is one of the enzymes that participates in DNA replication in prokaryotes [2,3].

Because of the great variety of DNA pols identified so far, they have been classified into six different families on the base of their aminoacidic sequence: A, B, C, D, X and Y. There is also a seventh family represented by reverse transcriptases (RTs), as they are also able to synthesize DNA [4].

DNA polymerase I belongs to the polymerase A family [5]; it is one of the most abundant DNA polymerases in prokaryotes [6,7,8]. It also possesses 3′–5′ exonuclease activity, for the excision of misincorporated nucleotides in order to ensure faithful and accurate synthesis of the new strand DNA [9], and 5′–3′ exonuclease activity which allows for the removal of deoxyribonucleotide and ribonucleotide primers (on the lagging strand) during DNA replication. The latter activity has also been described as 5′ nuclease or more commonly as flap endonuclease or FEN activity [10,11].

For many years, DNA polymerases have been used in the laboratory in molecular biology techniques for the in vitro DNA amplification [12]. Furthermore, many of them have been engineered for this use [13]. The most common technique for DNA amplification is PCR which has been widely used in the last 20–30 years for cloning but also for the identification of microorganisms in organic fluids (see molecular test for COVID) [14,15,16]. In fact, the direct DNA test on clinical samples can not only increase the sensitivity but, above all, simplify the analysis procedures.

In the early 1990s, new amplification techniques, generically known as isothermal amplifications, were developed by researchers who wanted to overcome the limitations of PCR, which requires expensive and complex equipment [17]. These techniques allow the synthesis of nucleic acids at a constant temperature, without the need of a thermal cycler, thus bringing an important innovation compared to the more common PCR. Among them, the loop-mediated isothermal amplification (LAMP) is one of the most promising, being characterized by its simplicity and reliability [18]. This technique is often used for point-of-care (POC) diagnostic testing [19,20,21].

LAMP uses the large fragment of DNA polymerase I from the thermophilic bacterium *Geobacillus stearothermophilus* (BstI DNA polymerase) that represents the counterpart of *E. coli* Klenow fragment; this enzyme, in addition to being endowed with polymerase activity, also possesses a DNA displacement activity that is necessary in isothermal amplification techniques, where nucleic acids are amplified without undergoing temperature variations [22].

LAMP technique uses a set of four to six primers that recognize six to eight regions of the target sequence. The primers form loop structures that facilitate the initiation of DNA synthesis and the generation of long, branched DNA products. The amplified DNA can be detected by fluorescence, colorimetry, or turbidity [23,24].

Despite the great advantage of the absence of temperature cycles, one of the important limitations remains the high temperature of the LAMP (63 °C), which means that this technique is still conditioned by the use of a thermal block.

In this context, we decided to identify a homologue of the BstI DNA polymerase Large Fragment from *Deinococcus radiodurans*, since it grows at 30 °C and is resistant to various environmental stress conditions. We demonstrated that the *D. radiodurans* DNA polymerase I Large Fragment (henceforth referred to as DraLF-PolI) displays efficient catalytic activity over a wide temperature and pH range and maintains its catalytic activity after exposure to high temperatures for several minutes, and the structural integrity of the protein was demonstrated with a Far-UV CD spectrum. Moreover, we found that DraLF-PolI possesses a strand-displacement activity starting from 10 up to 40 °C, even with a 1-nt gap or 0-nt gap, and retains its activities even under various stress conditions, such as exsiccation and long storage at 4 °C and room temperature. Finally, we found that DraLF-PolI is able to synthesize DNA even if one of the four nucleotides is missing from the mixture or only one of the four is provided.

The protein’s unique structural and functional properties make it a promising candidate for the development of a novel LAMP-based diagnostic platform, which could revolutionize point-of-care testing. This evolution could innovate the diagnosis of infectious diseases, enabling a faster and more practical detection of pathogens at the point of care.

## 2. Results

### 2.1. DraLF-PolI Displays Efficient Catalytic Activity over a Wide Temperature Range

**Figure 1:**
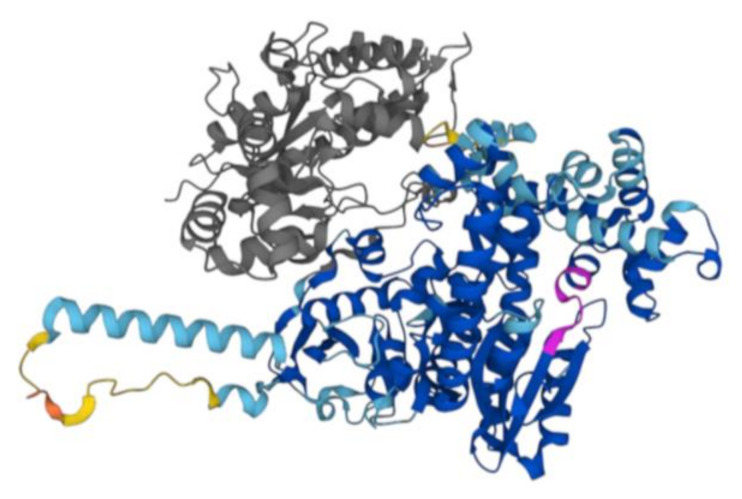
AlphaFold2 structure of *D. radiodurans* DNA Polymerase I. The Alphafold model of DNA polymerase I P52027 (DPO1_DEIRA) is reported in the figure. The poorly structured N-terminal region, aa 1–37, has been removed. In grey, the large region (38–361 aa) deleted in the construct used in our study including the nuclease domain and a partial 3′–5′ exonuclease domain is shown. In magenta, there is the evolutionarily conserved Motif A. The other colors are dictated by different confidence levels: dark blue: very high (pLDDT > 90); light blue: high (90 > pLDDT > 70); yellow: low (70 > pLDDT > 50); orange: very low (pLDDT < 50).

As shown in Figure 1, an Alphafold-model of DNA polymerase from *D. radiodurans* has been built. Based on this observation, we decided to clone this identified fragment and overexpress it in *E. coli* as described in Section 4. The protein was then purified to the homogeneity using Ni-NTA agarose column, reaching a yield of 5 μmol with a final concentration of 3 pmoles/μL (see Figure S1).

**Figure 2:**
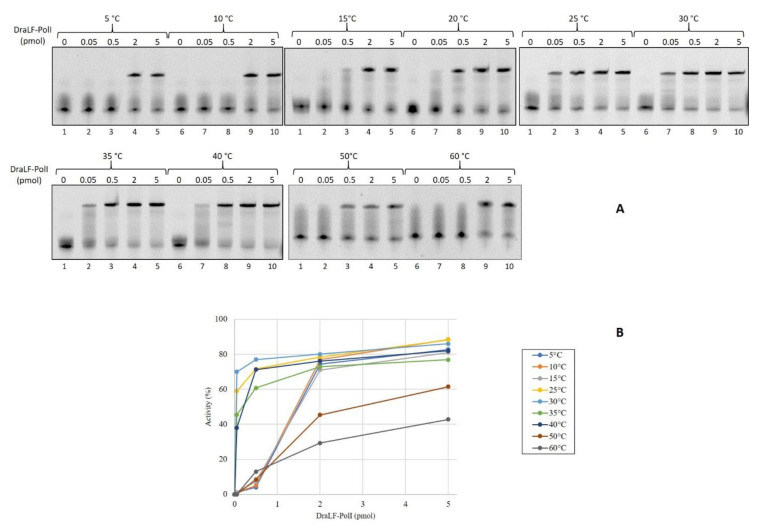
Analysis of DraLF-PolI DNA polymerase activity at different temperatures. (**A**) Increasing amounts of DraLF-PolI (from 0.05 up to 5 pmoles) were analyzed for their DNA polymerase activity on PE substrate (obtained as described in Section 4). The reactions were performed at the indicated temperatures. Lane 1 represents the control reaction where DraLF-PolI was omitted. (**B**) Graphic representation of the DNA polymerase assays at the indicated temperatures. The values obtained are the average of three different experiments.

First, we decided to verify if DraLF-PolI was able to amplify the DNA at room temperature with the aim of using it in a device for isothermal amplification detection at the point of care. Since temperature can be very inconstant and variable depending on the latitudes, we tested DNA polymerase activity at different incubation temperatures ranging from 5 °C to 60 °C, using increasing amounts of enzyme, from 0.05 up to 5 pmoles so to obtain a complete characterization of the enzyme. As shown in Figure 2, DraLF-PolI displays its polymerase activity over the entire temperature range tested, with a maximum efficiency between 25 and 40 °C. As can be clearly seen from the graph shown in Figure 2, panel B, when 5 pmoles of DraLF-PolI were used, the polymerase activity went from 40 to 70% in the range between 25 °C and 40 °C.

### 2.2. DraLF-PolI Retains Its Catalytic Activity after Exposure to High Temperatures

**Figure 3:**
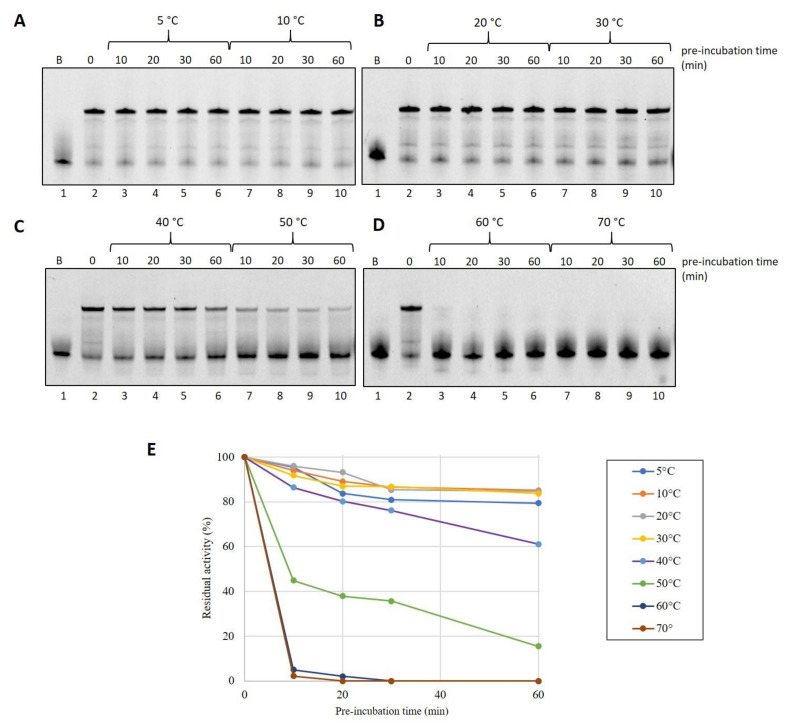
Thermostability analysis of DraLF-PolI. In total, 3 pmoles of of DraLF-PolI were preincubated at the indicated different temperatures for increasing times (from 0 up to 60 min) (**A**–**D**). The residual activity of the enzyme was tested at 30 °C on PE substrate after 10, 20, 30 and 60 min (lanes 3–6 and 7–10). Lane 1 refers to the negative control in which the substrate was incubated for 60 min in the absence of protein. Lane 2 is the positive control where the protein was not preincubated. (**E**) Graphic representation of DraLF-PolI thermostability at the indicated temperatures. The values are the average of three different experiments.

To test the ability of the protein to withstand thermal stress that may occur during device storage conditions, we decided to carry out thermoresistance assays. In total, 3 pmoles of DraLF-PolI were pre-incubated for the indicated times, up to a maximum of 60 min, in a range of temperatures that spans from 5 °C to 60 °C, as indicated in Figure 3. The residual activity was then tested by DNA polymerase assays at 30 °C as described in Section 4.

Surprisingly, we found that DraLF-PolI, despite being a mesophilic enzyme, possesses a moderate thermostability, retaining 40% activity even after 30 min of exposure at 50 °C (see Figure 3, panels C and E).

On the contrary, DraLF-PolI completely lacks its activity after 10 min at 60 °C (see Figure 3, panel D and E). These observations might seem to conflict with the thermophilicity data reported in Figure 2, where the enzymatic activity is detectable even at 60 °C and with 0.05 pmoles. To gain a deeper insight into these data discrepancies, we performed a time-course at 60 °C, measuring the activity on very short time scales starting from 5 s up to 240 s.

**Figure 4:**
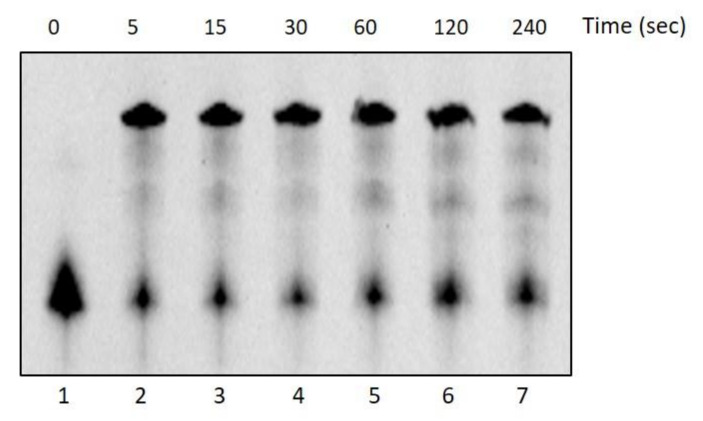
DNA Polymerase activity time-course at 60 °C. The activity of DraLF-PolI (3 pmoles) was tested at 60 °C, on a PE substrate, at increasing times, from 5 to 240 s (lanes 2–7). The negative control was performed in the absence of enzyme (lane 1).

As shown in Figure 4, the enzyme reaches its maximum activity after just 5 s of incubation (lane 2) before losing its stability and folding.

### 2.3. Structural Analysis of DraLF-PolI with Far-UV CD Spectrum

**Figure 5:**
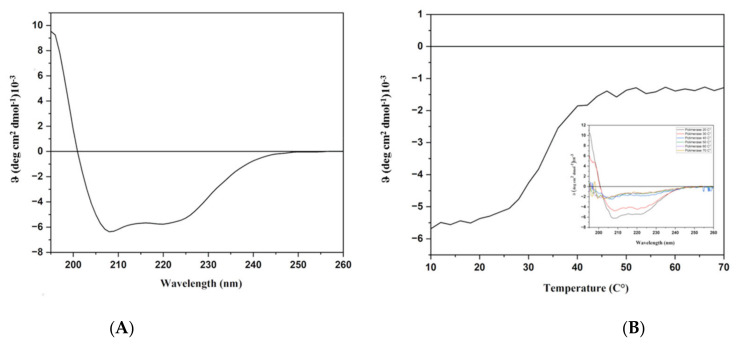
CD analyses. (**A**): Far-UV CD spectrum recorded at 20 °C. (**B**): Thermal denaturation monitored at 220 nm. In the insert, spectra registered at different temperatures.

In order to check the structural integrity of the protein sample, a Far-UV CD spectrum was registered. As expected, on the basis of the literature data on homologous proteins [25] and on the Alphafold model (Figure 1), the far UV CD spectrum was typical of a predominantly α-helical protein with two minima at 208 and 222 nm and a maximum around 190 nm. (Figure 5A). Therefore, under the experimental conditions used, the protein was proven to be folded. The thermal stability was investigated by far-UV CD spectroscopy and the Tm value derived from the temperature ramp denaturation curve following the CD signal at 220 nm is 35 °C. Nevertheless, the spectra registered at higher temperatures until 70 °C displayed a partial preservation of the secondary structure of the protein to justify activity at higher temperatures (Figure 5B).

The AlphaFold model produced showed a high model confidence score for residual, pLDDT > 90 and in particular in the entirety of the domain corresponding to the DNA polymerase I Large Fragment. In addition, the model showed that the evolutionarily conserved Motif A 735DYSQIELR 740, which is superimposable in all polymerases with resolved structures [26], as usual starts with an antiparallel hydrophobic β-sheet proceeding towards an α-helix.

### 2.4. DraFT-PolI Is Able to Continue DNA Synthesis Even in the Absence of the Correct Complementary Nucleotide

**Figure 6:**
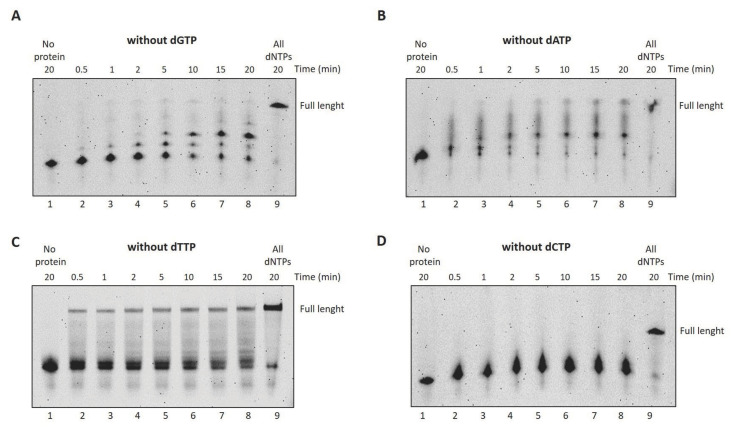
DraLF-PolI selectivity for dNTPs over time. (**A**–**D**) DNA polymerase assays without dGTP, dATP, dTTP and dCTP, respectively. In total, 3 pmoles of DraLF-PolI were incubated at 30 °C in the absence of one of the 4 dNTPs, at increasing times, as indicated (lanes 2–8). Lane 9 corresponds to the positive control in which the reactions were carried out in the presence of all dNTPs. Negative controls were performed in the absence of protein (lane 1).

In order to investigate the nucleotide selectivity of the enzyme, we performed DNA polymerase assays providing only three out of four nucleotides to the reaction [27]. As shown in Figure 6, the incorporation efficiency of the correct nucleotides is only partially compromised, especially when dTTP is the missing nucleotide; in this case, in fact, a full-length product can also be observed (see lanes 2–8, Panel C).

**Figure 7:**
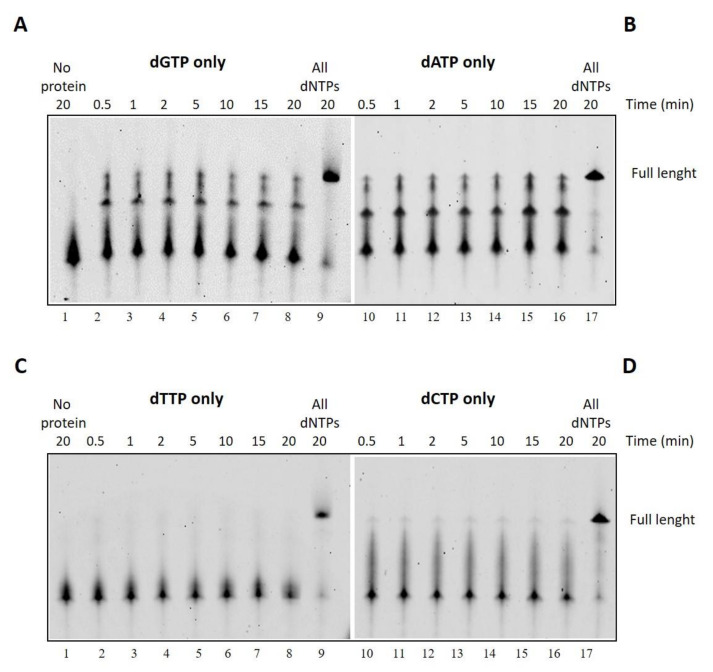
Incorporation of a single dNTP over time. (**A**–**D**) DNA polymerase assays with dGTP, dATP, dTTP or dCTP, respectively. In total, 3 pmoles of DraLF-PolI were incubated in the presence of only one of the four nucleotides, at increasing times, as indicated (lanes 2–8). Lane 9 correspond to the positive control in which the reactions were carried out in the presence of all dNTPs. Negative controls were performed in the absence of protein (lane 1).

We therefore decided to gain further insight and verify DraLF-PolI activity by providing only one of the four nucleotides. As can be seen in Figure 7, the synthesis activity of DraLF-PolI is drastically affected, but the enzyme is still able to incorporate nucleotides to the detriment of the complementarity.

### 2.5. DraLF-PolI Possesses a Strand-Displacement Activity

One of the key features of the DNA polymerase used in LAMP technology is the strand-displacement activity that allows the enzyme to displace downstream DNA encountered during synthesis so that it can proceed with its polymerization activity.

To investigate the ability of DraLF-PolI to perform strand displacement, we designed specific substrates that we called SDs (strand displacement substrates, see Section 4).

**Figure 8:**
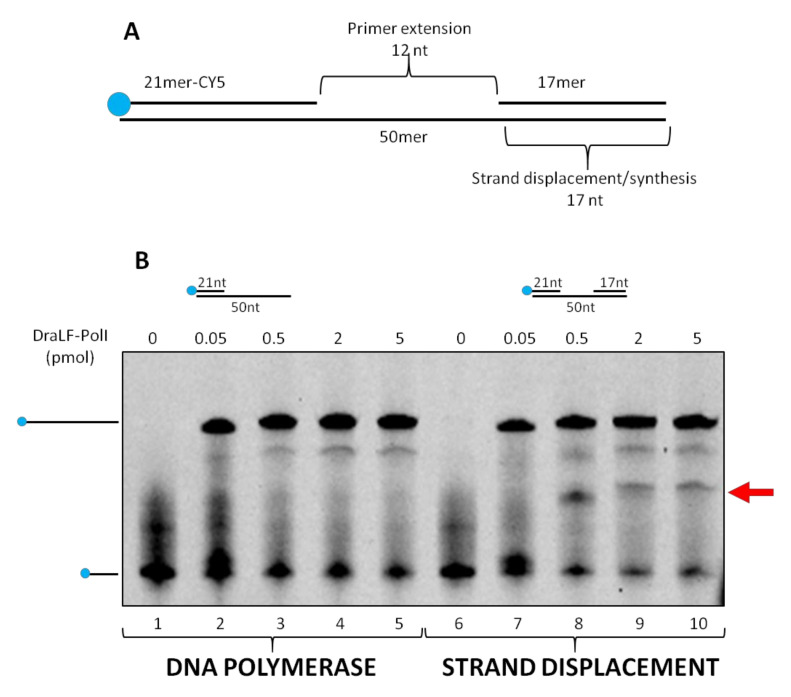
Strand-displacement activity assays. DraLF-PolI polymerase activity was tested on PE and SD substrates. (**A**) Schematic representation of the substrate used in this experiment, lanes 6–10. (**B**) DNA polymerase vs Strand-Displacement assays. A negative control was carried out in the absence of enzyme (lanes 1 and 6). Increasing amounts of DraLF-PolI (lanes 2–5; lanes 7–10) were analyzed.

The substrate was incubated for 10 min at 30 °C in the presence of increasing amounts of DraLF-PolI as indicated in Figure 8. In the absence of strand-displacement activity, primer extension should stop after the incorporation of twelve nucleotides, whereas strand-displacement activity should allow the enzyme to synthesize full-length products (50mer product).

As expected, we demonstrated that DraLF-PolI still retains strand-displacement activity despite being only a fragment of the entire enzyme. Indeed, as shown in Figure 8, a full-length product can be observed despite the block due to the presence of the 17mer oligonucleotide which is located 12 nucleotides downstream (Figure 8, lanes 7–10). Moreover, by comparing the primer extension (lanes 2–5) with the strand displacement (lanes 7–10) activities, we observed a different polymerization pattern; indeed, an intermediate product (indicated by the arrow, Figure 8) appears only when SD substrate was used. This intermediate product corresponds to the 21mer that is 12 nucleotides longer, indicating that the polymerase pauses before displacing the obstacle and continuing DNA synthesis. This observation confirms that DraLF-PolI retains its strand-displacement activity.

### 2.6. DraLF-PolI Displays Strand-Displacement Activity Even with a 1-nt Gap or 0-nt Gap

Therefore, we decided to analyze whether a minimum gap region was necessary for DraLF-PolI to perform its strand-displacement activity. To this aim, we designed two different substrates in which there was 1-nt or 0-nt gap between the first and second primers (as described in Section 4).

**Figure 9:**
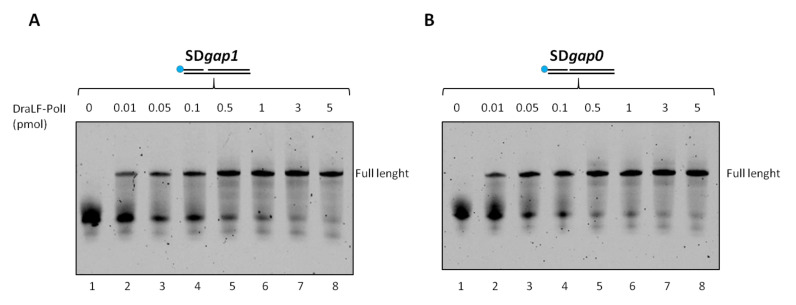
Strand-displacement activity assays of DraLFPolI on SDgap0 and Sdgap1 substrate. DraLF-PolI polymerase activity was tested on a substrate with 1 nucleotide gap (**A**) and on a substrate with no gap (**B**). Strand-displacement activity is observed in the presence of increasing quantities of DraLF-PolI (0.01 at 5 pmoles, as indicated); the positive controls (lanes 9, (**A**,**B**)) were performed on the PE substrate in order to unequivocally identify the full-length product (lanes 2–8, panels (**A**,**B**)). A negative control was performed in the absence of enzyme (lanes 1, panels (**A**,**B**)).

Increasing amounts of DraLF-PolI, from 0.01 to 5 pmoles, were incubated for 10 min at 30 °C in the presence of new constructs (see Section 4). DraLF-PolI shows strand-displacement activity in both cases indicating that the enzyme does not require a minimal gap region to perform displacement activity (Figure 9). These data confirm that DraLF-PolI does not require a minimal gap region for strand-displacement activity, but a nick on the leading strand is sufficient.

### 2.7. DraLF-PolI Exhibits Strand-Displacement Activity from 10 °C to 40 °C

Since LAMP technology requires both enzymatic activities (DNA polymerase and strand displacement), we decided to analyze the effect of the temperature on DraLF-PolI strand-displacement activity. We performed strand displacement assays as described above using increasing amounts of enzymes (0.01 to 5 pmoles) at different temperatures ranging from 10 °C to 40 °C.

**Figure 10:**
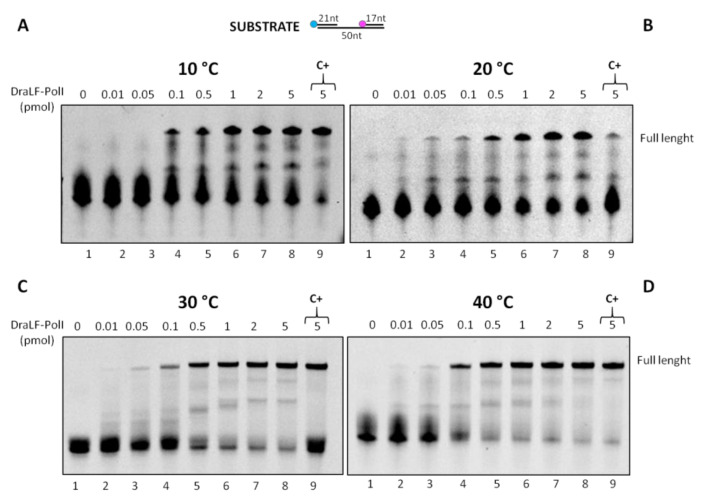
DraLF-PolI strand-displacement activity assays at increasing temperatures. The strand-displacement activity is observed in the presence of increasing amounts of protein (0.01 to 5 pmoles) (lanes 2–8, panels (**A**–**D**)). Negative controls were performed in the absence of proteins (lanes 1, panels (**A**–**D**)); positive controls (lanes 9, panels (**A**–**D**)) were performed with PE substrate. The temperatures analyzed are indicated.

As shown in Figure 10, the strand-displacement activity is detectable at all temperatures analyzed. Surprisingly, the activity is also detectable at 10 and 20 °C (Figure 10, panels A and B), where DNA melting is notoriously inhibited.

It is important to note that the temperatures analyzed correspond to the most diverse environmental conditions where a POC can be used, which vary depending on latitudes and seasons.

### 2.8. DraLF-PolI Binding to DNA Molecules

In order to further characterize DraLF-PolI biochemical properties, we decided to analyze its binding efficiency to different DNA structures. Increasing amounts of DraLF-PolI (from 0.5 to 40 pmoles) were incubated in the presence of various substrates, and the binding was analyzed as described in Section 4.

**Figure 11:**
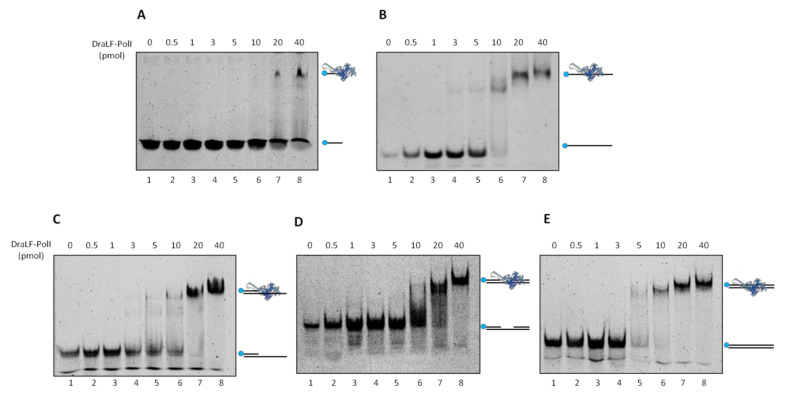
DNA binding affinity of DraLFPolI with different DNA structures. Electrophoretic mobility shift assays were performed using different DNA substrates as described in Methods to estimate binding affinity of DraLFPolI. Binding affinity to ssDNA 21mer (**A**) and 45mer (**B**); (**C**) binding affinity to a primed DNA structure; (**C**) binding affinity to a gapped dsDNA; and (**D**) binding affinity to a dsDNA. The assays were performed in the presence of increasing amounts of protein from 0.5 to 40 pmoles, as indicated (lanes 2–8, panels (**A**–**E**)). The negative controls were performed in the absence of protein (lane 1, panels (**A**–**E**)).

The results shown below (Figure 11) indicate that DraLF-PolI binds with comparable efficiency to all DNA molecules analyzed, regardless of whether they are single-stranded or double-stranded. The only exception is observed when the substrate used was only 21 nucleotides long (Panel A); in fact, in this case, the binding of the enzyme is visibly altered. Taken together, these data suggest that DNA length drastically influences the stability of DraLF-PolI binding to DNA.

### 2.9. DraLF-PolI Remains Fully Active over a Wide pH Range

To gain a deeper insight into the potential suitability of DraLF-PolI for the detection of DNA from microorganisms coming from the most diverse sources or from biological liquids, we decided to test the DNA polymerase activity in a pH range between 6.4 and 10. This choice is based on the fact that *D. radiodurans* can grow in a pH range from 6.4 to 8.0 [28].

**Figure 12:**
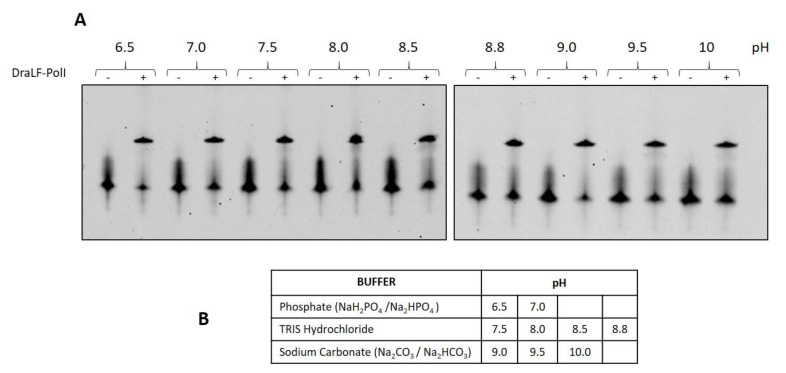
Analysis of DraLF-PolI activity at different pHs. (**A**) DNA polymerase activity of DraLF-PolI was evaluated at different pH. In total, 3 pmoles of enzyme were incubated in the presence of PE substrate as described in Section 4. (**B**) Selected buffers. The negative controls (-) were performed in the absence of protein.

As can be observed in Figure 12, DraLF-PolI maintains its activity throughout the pH range analyzed, supporting the possibility of its potential use in the most varied POC and from the most varied sources.

### 2.10. Analysis of DraLF-PolI Activity under Various Storage Conditions

With the hypothesis of using DraLF-PolI in an innovative LAMP device, we decided to test its residual activity following different storage conditions.

First, we decided to test the resistance of DraLF-PolI to desiccation since the bacterium *D. radiodurans* is able to survive this condition. To this aim, aliquots of enzyme were dried under high vacuum (Eppendorf concentrator 5301 system), and to evaluate the effect of desiccation on the enzyme activity, we first performed a zero-time test with increasing amounts of rehydrated protein. The results indicate that drying only partially reduces DNA polymerase activity (see Figure S2), which prompted us to test its residual activity over time, after storage at room temperature.

**Figure 13:**
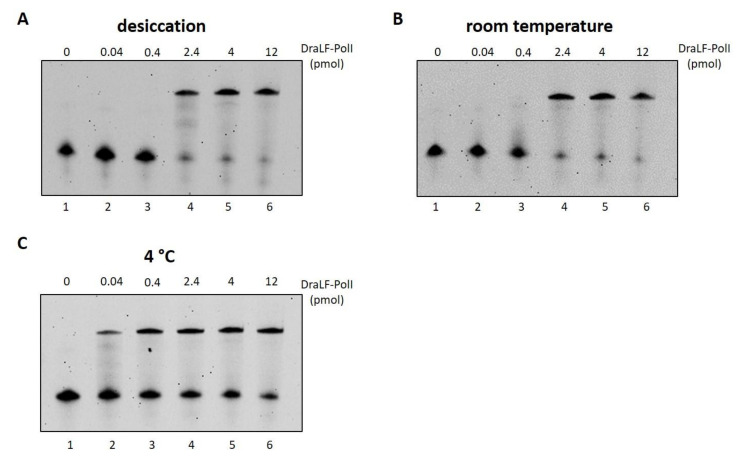
Analysis of DraLF-PolI activity under different storage conditions. DraLF-PolI polymerase activity was tested on PE substrate as described in Section 4, under different storage conditions. (**A**) Residual activity 24 h after desiccation, storage at room temperature and rehydration; (**B**) residual activity 24 h after storage in a liquid state at room temperature; (**C**) residual activity 24 h after storage in a liquid state at 4 °C. The assays were performed in the presence of increasing amounts of protein from 0.04 to 12 pmoles, as indicated (lanes 2–6, panels (**A**–**C**)). The negative controls were performed in the absence of protein (lane 1, panels (**A**–**C**)).

In addition to desiccation, we stored the enzyme in a liquid state at room temperature and at 4 °C and tested the residual activities after 24 h with increasing amounts of enzyme (Figure 13, panels A, B and C). DraLF-PolI remains always active, and in particular, at 4 °C, only 0.04 pmoles are sufficient to obtain a full-length product, suggesting that this is the best storage condition within 24 h.

Interestingly, if we compare the activity of the protein immediately after drying (Figure S1) with that obtained from the protein dried and stored at room temperature for 24 h (Figure 13, Panel A), it is clear that the activity remains constant over time, indicating that the loss of activity is related only to the drying process and not to the storage conditions. These data suggest that drying represents a practical and functional storage method for DraLF-PolI.

## 3. Discussion

DNA replication is a process that occurs in all living organisms and is the basis of biological inheritance. The main players in this process are DNA polymerases, which have been used in various methodologies where precise and faithful amplification of DNA is essential. Among these, the most widespread technology is the polymerase chain reaction (PCR), a technique that allows for the exponential amplification of specific DNA fragments with high sensitivity and specificity. The basis of PCR is a thermophilic DNA polymerase, which acts at high temperatures (generally 72 °C) and involves several steps for DNA amplification, each characterized by a specific temperature. Therefore, this methodology requires not only a thermal cycler necessary for the automation of the process, but also specialized personnel able to set up the procedure and analyze the data. To overcome the limitations of PCR in the 1990s, new amplification techniques were developed [29], which allow the synthesis of nucleic acids at a constant temperature, without the need of thermal cyclers. This new technology, known as isothermal amplification, has had extensive development, giving rise to multiple amplification techniques; among these, the most promising is the loop-mediated isothermal amplification (LAMP) [30,31]. This technique involves the use of Bst PolI, a DNA polymerase that, in addition to having synthetic activity, also has strand-displacement activity, indispensable for eliminating the DNA re-opening phase used in PCR during the amplification process.

LAMP has proven particularly cutting-edge in the healthcare and food sectors, where rapid detection of contaminating DNA is necessary, but complex and specialized equipment, such as thermal cycler, as well as trained personnel, are not easily available. For the correct functioning of the LAMP, it is however necessary to use a thermal incubator to reach the constant temperature of 65 °C necessary for the functioning of Bst PolI.

The aim of this work is to identify a DNA polymerase homologous to Bst PolI, which works at room temperature. We therefore decided to use the polyextremophilic bacterium *D. radiodurans* as an enzyme source, since it grows at 30 °C, and its enzymes are characterized by high resistance to extreme conditions, such as high doses of genotoxic agents, ionizing radiation, desiccation and oxidative stress. Through sequence alignment, we then identified the genetic sequence encoding the Bst PolI homolog and cloned the region corresponding to the BstI large fragment. Once the enzyme, referred to as DraLF-PolI (*D. radiodurans* Large Fragment—Polymerase I), was expressed and purified to homogeneity, we conducted a functional and structural characterization, with the aim of demonstrating that this enzyme can really be an excellent candidate for a possible improvement of LAMP technology, a system of great interest today but which nevertheless presents some limitations. Our results demonstrated that DraLF-PolI retains both DNA polymerase and strand-displacement activities, functions over a wide range temperature from 10 °C to 40 °C with an optimum between 25 °C and 35 °C. These results suggest that the use of DraLF-PolI would make the LAMP technology stable in the most varied environmental conditions and thus avoiding the use of a thermal block. Furthermore, we demonstrated that DraLF-PolI strand-displacement activity is present even in the absence of a single-stranded region (gap) between the newly synthesized product and the substrate to be opened. This suggests that the strand-displacement activity is robust and does not slow down the synthesis activity of the enzyme. We also demonstrated that DraLF-PolI can bind to various DNA structures, but the binding region must be longer than 20 nucleotides for stable binding. Moreover, we showed that DraLF-PolI is able to synthesize DNA even in the presence of only one nucleotide and it maintains its activity in a wide pH range, and since our goal is to identify microorganisms from the most diverse sources or biological liquids, this adaptability makes it even more suitable to our research purposes. Finally, we tested the resistance of DraLF-PolI to various storage conditions with the aim of identifying the most suitable ones in a cutting-edge LAMP system for use in POCs. We demonstrated that the enzyme is still able to polymerize DNA after long periods of storage at room temperature and at 4 °C, but the best storage condition is desiccation.

All these characteristics, taken together, make DraLF-PolI an excellent candidate to exploit for a room-temperature LAMP that promises to be very useful for the rapid and simple detection of pathogens at the POCs.

## 4. Material and Methods

### 4.1. Protein Structure Prediction

The three-dimensional structure of DraLF-PolI was built on the basis of the primary sequence using Alphafold methodology [32,33].

### 4.2. Cloning of DraLF-PolI Encoding Gene

With the aim to identify the homolog of the BstI Large Fragment in *D. radiodurans* sequence, an alignment between *D. radiodurans* PolI (I P52027) and *Geobacillus stearothermophilus* DNA PolI (D 9N168) was performed by Clustal Omega [https://www.ebi.ac.uk/Tools/msa/clustalo/ (accessed on 1 May 2022)]. Based on the bioinformatic results, we decided to clone the amino acid region 362–956. The codon-optimized genes, encoding DraLF-PolI gene, was purchased from the GenScript Synthesis service [GenScript Biotech (Amsterdam, The Netherlands) B.V.]. The gene was cloned into the expression plasmid pET-29b(+) between the NdeI and HindIII restriction sites, encoding a C-terminal His6-tag.

### 4.3. Expression and Purification of Recombinant Proteins

*E. coli* BL21 (DE3) cells (Novagen, Madison, WI, USA) transformed with the plasmid pET29b-DraLF-PolI were grown at 37 °C in 500 mL of LB (Luria–Bertani) medium containing 30 μg/mL kanamycin. When the culture reached an A_600_ of 0.6 OD, protein expression was induced by adding isopropyl β-d-thiogalactoside (IPTG) at a concentration of 0.5 mM. The bacterial culture was incubated overnight at 30 °C. Cells were then harvested by centrifugation (8.000 rpm Beckman centrifuge, rotor JA 14) and the pellet (5 g) was stored at −20 °C until use. The cell pellet (5 g) was thawed and resuspended in 25 mL of buffer A (20 mM Tris/HCl pH 8.0, 500 mM NaCl, 3 mM β-mercaptoethanol, 5% glycerol, 10 mM imidazole) supplemented with protease inhibitor cocktail (Complete Mini EDTA-free, Roche, Basil, Switzerland) and lysed with sonication (30 s 10 Hz and 30 s pause, 8 times). The resulting cell extract was treated with DNase I (at 0.25 mg/mL) and RNase A (at 0.1 mg/mL) for 30 min on ice with shaking. The sample was centrifuged for 30 min at 20,000 rpm (Beckman centrifuge, rotor JA 14) at 10 °C. The supernatant was passed through a 0.22 μm filter (Millipore, Burlington, MA, USA), mixed with 0.5 mL of Ni^2+^-nitrilotriacetic acid agarose resin (Qiagen, Hilden, Germany), pre-equilibrated in buffer A and incubated for 1 h on ice with gentle shaking. The resin was washed with buffer A, and elution was carried out with a step gradient of imidazole (from 0.1 M to 1 M) in buffer A. Fractions were analyzed with SDS/PAGE (see Figure S1), the peak fractions, eluted at 100 mM imidazole, were pooled and dialyzed overnight at 4 °C against storage buffer containing 20 mM Tris/HCl pH 8.0, 100 mM NaCl, 5% glycerol.

### 4.4. Substrate Preparation

All of the oligonucleotides used were synthesized by Eurofins Genomics srl, and their sequences are listed in Table 1.

**Figure 14:**
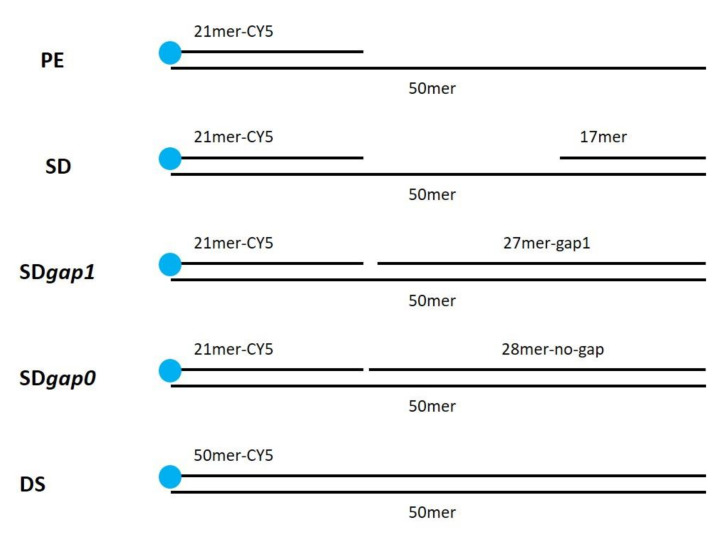
Substrate structure used in this work.

The DNA polymerase substrates were generated by annealing the oligonucleotides as described below (see Figure 14).

PE (pimer extension substrate) was obtained by annealing equimolar amounts of 50mer and 21mer-Cy5; SD (strand-displacement substrate) was obtained by annealing equimolar amounts of 50mer, 21mer-Cy5 and 17mer; SDgap1 was obtained by annealing equimolar amounts of 50mer, 21mer-Cy5 and 27mer-gap1; SDgap0 was obtained by annealing equimolar amounts 50mer, 21mer-Cy5 and 28mer-no-gap. DS (double strand) was obtained annealing equimolar amounts of 50mer and 50mer-Cy5.

The oligonucleotides were incubated for 5 min at 95 °C and then slowly cooled at room temperature in order to obtain the indicated structures.

### 4.5. DNA Polymerase and Strand Displacement Assays

To perform the DNA polymerase assay, PE (primer extension substrate), generated as previously described (see Section 4.4 and Table 1), was used. To perform the strand displacement assay, SD (strand-displacement substrate), generated as previously described (see Section 4.4 and Table 1), was used.

The reaction mixture (10 μL) contained 50 mM Tris/HCl pH 8.8, 1 mM DTT, 0.05% Triton-100, 10 mM MgCl_2_, 1 mM dNTPs and 50 nM labeled substrate (PE or SD). Reactions were initiated by adding the indicated amounts of DraLF-PolI and incubated at 30 °C for 10 min. Reactions were stopped by adding 5 μL of Stop Solution (90% formamide, 50 mM EDTA) heated at 95 °C for 5 min and briefly cooled on ice.

The polymerized products were analyzed by electrophoresis in a 12.5% polyacrylamide gel (acrylamide/bis-acrylamide 19:1) containing 6 M urea, 1X TBE and visualized by VersaDocTM MP 1000 system (Bio-Rad, Hercules, CA, USA). ImageJ 1.5 software was used for quantitative analysis. All experiments were repeated three times.

### 4.6. Circular Dichroism

CD spectrum of DraLF-PolI was recorded at 20 °C in the far UV (190–260 nm) on a Jasco J-1500 spectropolarimeter (Tokyo, Japan) using a quartz cell with 0.1 cm path length. Each spectrum was acquired averaging three scans, subtracting contributions from corresponding blanks and converting the signal to mean residue ellipticity in units of degree cm^2^ dmol^−1^, using the following parameters: 0.5 nm data pitch, 20 nm/min scanning speed, 4 s of D.I.T. and 1 nm spectral band width. Spectra were recorded using 6 μM DraLF-PolI in 20 mM Tris/HCl pH 8.8, 50 mM NaCl, 5% Glycerol [34]. Thermal denaturation studies were carried out following signal CD at 220 nm from 20 °C to 70 °C with an increase of 1 °C/min. Spectra were registered every 10 °C.

### 4.7. Electrophoretic Mobility Shift Assay, EMSA

For each substrate, reactions were performed in a final volume of 10 µL containing the indicated amounts of protein and 0.05 μM of different labeled DNA substrates in 1× Binding Buffer (20 mM Tris/HCl, pH 7.5, 3.5 mM β-mercaptoethanol, 5 mM MgCl_2_, 2 mM EDTA, 50 mM NaCl). Following incubation for 10 min at room temperature, 1 µL of 100% glycerol was added, and the complexes were separated by electrophoresis through 5% polyacrylamide (29:1) gels in 0.5 × TBE. The products were visualized by a VersaDocTM MP 1000 system (Bio-Rad).

## Figures and Tables

**Table 1:** Sequences of the oligonucleotides used in this work.

Substrate	Oligonucleotide Sequence
21mer-CY5	5′-CY5-GCTATCGTACATGATAT CCTC-3′
50mer	5′-GTAGTGACTGGGAAAACATGTACGATAGCGAGGATATCATG TACGATAGC-3′
17mer	5′-GTTTCCCCAGTCACGAC-3′
27mer-gap1	5′-CTATCGTACATGTTTTCCCAGTCACTAC-3′
28mer-no-gap	5′-GCTATCGTACATGTTTTCCCAGTCACTAC-3′
50mer-CY5	5′-CY5-GCTATCGTACATGATATCCTCGCTATCGTACATGTTTTCC …CAGTCACTAC-3′

## Data Availability

The original contributions presented in the study are included in the article/Supplementary Materials, further inquiries can be directed to the corresponding author/s.

## Supplementary Materials

The following supporting information can be downloaded at: https://www.mdpi.com/article/10.3390/ijms25031392/s1.
