# Supplementary material for: DNA Polymerase I Large Fragment from Deinococcus radiodurans, a Candidate for a Cutting-Edge Room-Temperature LAMP
Source: Int J Mol Sci. 2024 Jan 23;25(3):1392. doi: 10.3390/ijms25031392 (PMC10855757; doi:10.3390/ijms25031392)
Supplement: Supplementary file 1 [file ijms-25-01392-s001.zip › ijms-2812143-supplementary.pdf]

DNA Polymerase I Large Fragment from *Deinococcus radiodurans*, a Candidate for a Cutting-Edge Room Temperature LAMP

Marilena Manzo[1, †], Assunta Serra[1, †], Emilia Pedone[2], Luciano Pirone[2], Viviana Scognamiglio[3], Mariarita De Felice[1,*,†] and Mariarosaria De Falco[1,*,†]

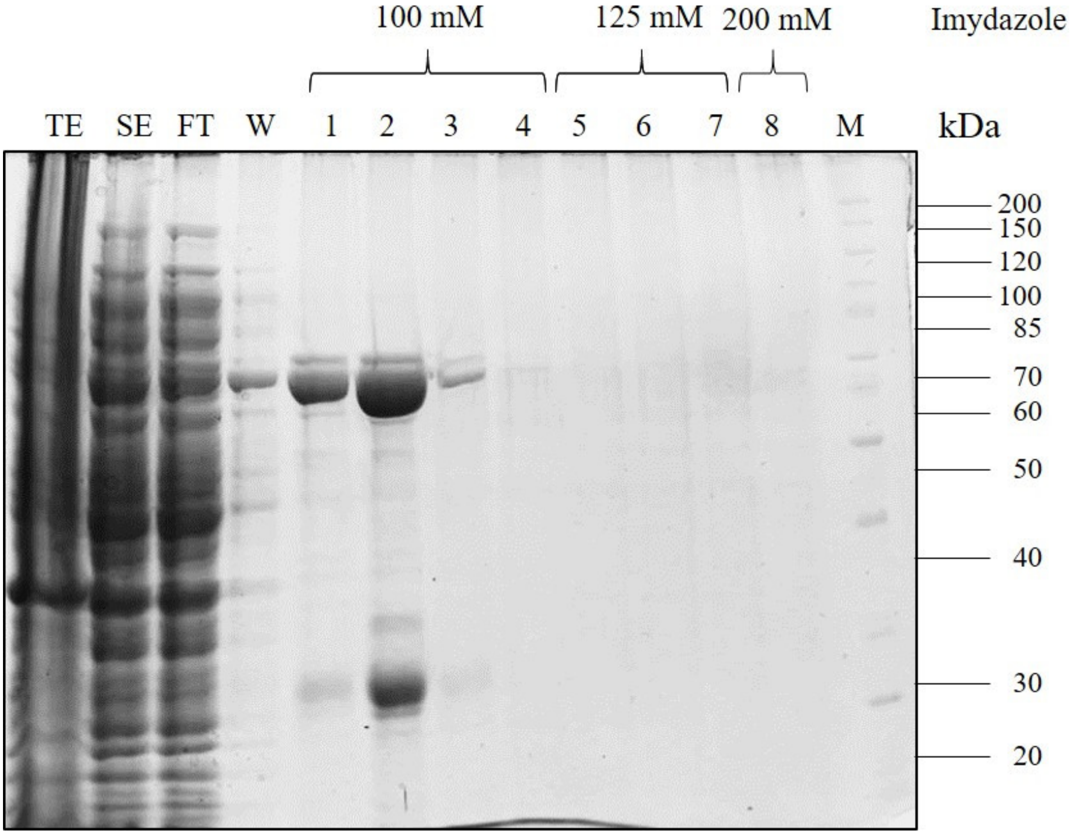

Figure S1. Nichel-NTA purification of DraLF-PolI. SDS-polyacrylamide gel of electrophoretic profile purification on Nichel-NTA resin. Lane TE (Total Extract); lane SE (Soluble Extract): lane W (Wash); lanes 1, 2, 3, 4 (100 mM Imydazole elution fractions); lanes 5, 6, 7 (125 mM Imydazole elution fractions); lane 8 (200 mM Imydazole elution fraction); lane M (molecular weight marker)

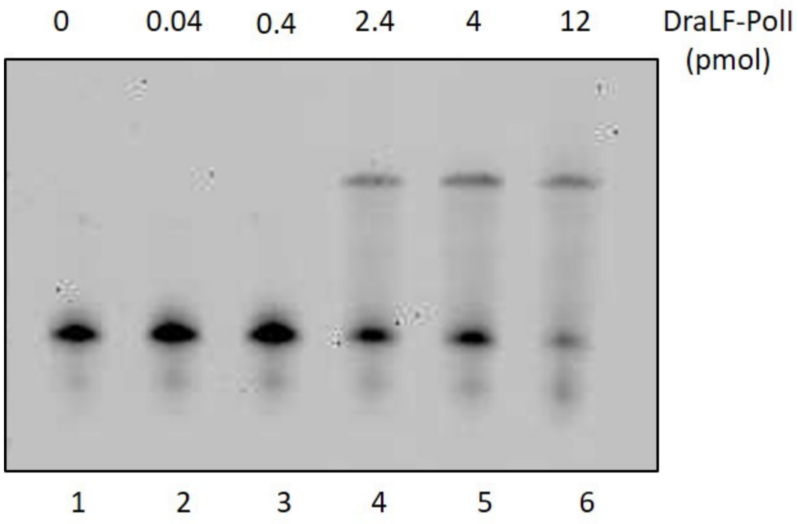

Figure S2. DraLF-PolI residual activity immediately after desiccation. DraLF-PolI polymerase activity was tested on PE substrate as described in Material and Methods. The assays were performed in the presence of increasing amounts of protein from 0.04 to 12 pmoles, as indicated (Lanes 2-6). The negative control was performed in the absence of protein (Lane 1).
